# Associations of Serum 25-Hydroxyvitamin D with Adiposity and At-Risk Lipid Profile Differ for Indigenous (Orang Asli) Male and Female Adults of Peninsular Malaysia

**DOI:** 10.3390/ijerph17082855

**Published:** 2020-04-21

**Authors:** Ee Yin Chua, Zalilah Mohd Shariff, Norhasmah Sulaiman, Geeta Appannah, Heng Yaw Yong

**Affiliations:** Department of Nutrition and Dietetics, Faculty of Medicine and Health Sciences, University Putra Malaysia, Serdang 43400, Selangor Darul Ehsan, Malaysia; eeyinchua@gmail.com (E.Y.C.); norhasmah@upm.edu.my (N.S.); geeta@upm.edu.my (G.A.); yong_hy@upm.edu.my (H.Y.Y.)

**Keywords:** vitamin D, adiposity, lipid profile, indigenous peoples

## Abstract

Background: Low vitamin D status, adiposity, and at-risk lipid profile are associated with adverse health consequences. This study aimed to assess serum 25(OH)D concentration of Indigenous (Orang Asli) adults and to determine the associations between serum 25(OH)D with adiposity and lipid profile, respectively. Methods: This cross-sectional study was conducted among 555 (164 men, 391 women) Orang Asli adults aged 18–65 years of Jah Hut sub-tribe in Krau Wildlife Reserve (KWR), Peninsular Malaysia. Demographic and socio-economic information were obtained using interviewer-administered questionnaire. Participants were also assessed for serum 25-hydroxyvitamin D (25(OH)D) concentration, adiposity indices (BMI, WC, WHtR, WHR, %BF) and lipid parameters (TC, LDL-C, HDL-C, TG). Data were analyzed using binary logistic regression via SPSS. Results: The prevalence of suboptimal 25(OH)D concentration was 26.3%, comprising 24.9% insufficiency (50 to <75 nmol/L) and 1.4% deficiency (<50 nmol/L). While men (14–30.5%) were associated with a more proatherogenic lipid profile than women (6.1–14.3%), more women were with central obesity (M: 19.5–46.3%; F: 34.5–49.1%) and suboptimal (<75 nmol/L) vitamin D status (M: 11.6%; F: 32.4%). While suboptimal 25(OH)D concentration was significantly associated with higher odds of at-risk LDL-C (*p* < 0.01) and obesity (WC, WHtR) (*p* < 0.05) in men, no significant association was observed for women. Nonetheless, it should be noted that there were only 19 men with suboptimal (<75 nmol/L) vitamin D status. Conclusions: While suboptimal vitamin D status was relatively low in Orang Asli adults, the prevalence of obesity and undesirable serum lipids were relatively high. The sex-specific associations between vitamin D status with adiposity indices and serum lipids warrant further investigation.

## 1. Introduction

Vitamin D is not only involved in bone homeostasis but also plays a role in various physiological functions of the body. As the main circulating form of vitamin D, serum 25-hydroxyvitamin D (25(OH)D) is used as a biomarker to evaluate vitamin D status [1]. Given foods naturally rich in vitamin D are few, sunlight exposure becomes the main source of vitamin D to human [2]. Lifestyle factors such as limited outdoor activity may reduce the opportunity of getting sufficient sun exposure for cutaneous vitamin D synthesis [3]. Female and dark skin are among the non-modifiable risk factors for low level of 25(OH)D [4].

Vitamin D deficiency is pandemic, and a multitude of potential adverse health consequences are attributed to low vitamin D status [5,6]. Studies have suggested an inverse association between 25(OH)D and cardiovascular risk biomarkers, including an atherogenic lipid profile [7,8]. Vitamin D regulates the signaling pathways that link both the uptake and clearance of cholesterol in macrophages in diabetic patients [9]. Vitamin D may also attenuate inflammation [10], which is integral to the development of cardiovascular diseases (CVD) [11].

While obesity is one of the major risk factors for CVD [12], vitamin D deficiency and obesity seem to be related occurrences. Obese individuals are more likely to have lower 25(OH)D [13]. Increased adipose tissues in the obese state will expand the distribution of the pool of vitamin D (since vitamin D is fat soluble), thereby reducing the circulating 25(OH)D concentration [14]. A recent bi-directional Mendelian Randomization approach to investigate the causal relationship between obesity and vitamin D deficiency has shown that higher BMI leads to lower vitamin D status [15]. A study in Korean population indicated that serum 25(OH)D concentration was independently associated with total body fat content but it may not be associated with the indicators for estimating adiposity, such as WC or BMI, after adjusting for confounders [15]. Several previous cross-sectional studies [13,16] showed that the direction of vitamin D—obesity relationship is uncertain as it could be subjected to different measures of adiposity. Measurements of adiposity in aforementioned studies have been limited to indirect anthropometric measures which may not consistently reflect the true adiposity in different populations.

The effect of vitamin D on lipid profile may be mediated by adiposity where adiponectin may be the key player in the mechanism. A positive influence of vitamin D on adiponectin levels has been reported [17,18]. Adiponectin could reduce plasma triglyceride by increasing VLDL triglyceride catabolism [19] and inhibit lipid accumulation in macrophage foam cells [20]. Given that adiponectin expression is reduced in obese subjects [21] and abdominal obesity may significantly influence the circulating adiponectin levels [22], the impact of vitamin D on lipid profile may vary across adiposity and its distribution.

Worldwide, vitamin D deficiency is prevalent. A quarter of Asian adults had 25(OH)D insufficiency (21–29 ng/mL) and almost half were with 25(OH)D deficiency (<20 ng/mL) [4]. Despite living in sun-rich environment, Malaysians are also at risk of low vitamin D status. Several local studies reported that at-risk groups including children, women of childbearing age and postmenopausal women have consistently shown a high prevalence of low vitamin D status [23,24,25,26,27]. Besides the at-risk groups, suboptimal vitamin D status is also prevalent among adult men in Malaysia [3].

The indigenous peoples of Peninsular Malaysia, also known as Orang Asli, have dark skin and high prevalence of cardio-metabolic risks [28]. To date, there is no published information on vitamin D status and its relationship to cardio-metabolic risks in this Orang Asli population. The present study assessed serum 25(OH)D concentration of Orang Asli adults and determined the associations between serum 25(OH)D with adiposity and lipid profile, respectively.

## 2. Materials and Methods

### 2.1. Study Design and Subjects

This cross-sectional study was conducted in Krau Wildlife Reserve (KWR), Peninsular Malaysia. Indigenous (Orang Asli) adults aged 18–65 years of Jah Hut sub-tribe, from 9 villages (out of 11) in KWR, were invited to participate in the study. Approximately 1500 Orang Asli adults were identified and one-third of them (555) were eligible and willing to participate in the study. Individuals were excluded from the study if they were pregnant, on vitamin D or calcium supplementation or diagnosed with endocrine disorders or liver/kidney disease (referred to hospital/clinic card).

The study was conducted in accordance with the Declaration of Helsinki, and the protocol was approved by the Medical Research Ethics Committee of the Faculty of Medicine and Health Sciences, Universiti Putra Malaysia (FPSK(FR14)P010). Permission to conduct this study was also obtained from the Malaysia Department of Orang Asli Development (JAKOA) and the Department of Wildlife and National Parks of Peninsular Malaysia (PERHILITAN). Prior to data collection, initialized or verbal informed consent was obtained from study respondents.

### 2.2. Anthropometeric Measurements

Weight and height were measured using TANITA HD-314 digital weighing scale and SECA 213 portable stadiometer, respectively. Hip and waist circumferences were measured using SECA 201 measuring tape. Percentage body fat (%BF) was determined using OMRON HBF-302 portable body fat analyzer. Body Mass Index (BMI), waist–hip ratio (WHR), and waist–height ratio (WHtR) were computed and categorized based on the recommendations of the World Health Organization (2000), International Diabetes Federation (2006), Browning, Hsieh, and Ashwell (2010), respectively [29,30,31]. The classification of percentage BF was based on Nieman (2003) [32].

### 2.3. Laboratory Measurements

Fasting venous blood (5 mL) of respondent was drawn and placed into a gold-top (serum separator) tube by a clinic nurse. Blood samples were stored at 2–8 °C using cold packs and transported to an accredited private laboratory on the same day for analyses of 25(OH)D and lipids. The blood samples were allowed to clot and centrifuged at 1100–2000× g for a minimum of 10 min, allowing the clear serum to be removed for testing. Serum 25(OH)D concentration and serum lipids (total cholesterol (TC), high density lipoprotein cholesterol (HDL-C), and triglycerides (TG)) were determined using one step, electrochemiluminescence based immunoassay and enzymatic method, respectively. All the above analyses were performed with Architect Ci8200 analyzer (Abbott Diagnostics, Chicago, IL, USA). The Friedewald equation was used to calculate low density lipoprotein cholesterol (LDL-C) in those with TG levels <4.52 mmol/L [33]. All lipid values were expressed as mmol/L. Serum 25(OH)D concentration was categorized as: sufficiency (≥75 nmol/L), insufficiency (50 to <75 nmol/L), and deficiency (<50 nmol/L) based on the Endocrine Society Clinical Practice guideline [34]. Lipid parameters (TC, TG, HDL-C, and LDL-C) were categorized based on NCEP ATP III (2001) classifications [35].

### 2.4. Statistical Analaysis

Analyses were performed using IBM® SPSS® 21 (IBM Corp., Armonk, NY, USA). All data except TG was normally distributed. The differences in the parameters according to the category of 25(OH)D concentration (25(OH)D concentration <75 nmol/L vs. ≥75 nmol/L) were determined by independent t-test and Mann–Whitney test. Sex-specific logistic regressions were performed to determine the association between status of 25(OH)D concentration with obesity status and lipid profile, respectively, using crude, age- and multivariable adjusted models. Lifestyle factors were among the covariates that were included in the multivariable adjusted models. Significant level was set at *p* < 0.05.

## 3. Results

### 3.1. Characteristics of Respondents

This study comprised 164 (29.5%) men and 391 (70.5%) women with a mean age of 37.6 ± 13.1 years (Table 1). About one third (33.7%) of Orang Asli adults had no formal education and 53.5% worked as rubber tappers. Only a minority (<5%) still practiced hunting and gathering on daily basis. Compared to women (4.6 h), men (6.8 h) spent more hours in the sun daily (*p* < 0.05). Men had higher (99.65 ± 23.32 nmol/L) mean 25(OH)D concentration than women (85.28 ± 21.54 nmol/L) (*p* < 0.001).

As shown in Table 2, the prevalence of suboptimal 25(OH)D concentration was 26.3%, comprising 24.9% insufficiency and 1.4% deficiency. A significantly larger proportion of women (32.4%) were with suboptimal 25(OH)D concentration compared to men (11.6%) (*p* < 0.001). About 34.2% of men and 37.4% of women were overweight or obese. At least one-third of Orang Asli adults were centrally obese. As compared to men, more women were with at risk WC (M: 19.5%; W: 34.5%, *p* < 0.05), WHtR (M: 44.5%; W: 56.0%, *p* < 0.05) and WHR (M: 46.3%; W: 49.1%, *p* > 0.05). The proportions of men and women with high percentage of body fat were similar (31.7%). Approximately 10% of adults were with high TC, LDL-C, and low HDL-C while one-fifth had high TG. Compared to the female adults, there were more than twice the proportion of male adults with high TG, TC, LDL-C, and low HDL-C (*p* < 0.05).

The characteristics of respondents according to 25(OH)D concentration are presented in Table 3. Comparing between adults who had suboptimal and sufficient 25(OH) D concentration, significant differences in adiposity measures (WC, WHtR, and %BF) were reported in men. Meanwhile, women in the two categories of 25(OH)D concentration had significant differences in TG, HDL-C, and LDL-C levels. There was no significant difference in WHR between the two groups in men and women whereas a significant higher TC level was found in adults who had suboptimal 25(OH)D concentration as compared to their counterparts.

### 3.2. Associations Between 25(OH)D Status and Obesity

Table 4 shows the associations between 25(OH) D status and obesity in men and women, respectively. Men with suboptimal 25(OH)D concentration were three times and about four times more likely to have at-risk WC and WHtR. The associations remained significant even after adjusting for age and other covariates. On the other hand, all the associations between 25(OH)D status and obesity in women were not significant. As shown in Table 5, compared to those with sufficient 25(OH)D concentration, men with suboptimal 25(OH)D concentration were four to five times more likely to have high LDL-C (*p* < 0.05) (Model 2, 3, and 4). Meanwhile, men with suboptimal 25(OH)D concentration were less likely to have high TG than their counterparts after adjusting for adiposity, particularly WC but the association is not significant (*p* > 0.05).

## 4. Discussion

The present study showed that the associations between 25(OH)D concentration and adiposity varied by sex and obesity indicators. Orang Asli male adults with suboptimal 25(OH)D status were significantly more likely to have central obesity as defined by at-risk WC (≥90 cm) and WHtR (≥0.5), independent of age and lifestyle factors. Lifestyle factors such as physical inactivity and smoking are associated with vitamin D deficiency and obesity in men, respectively [36,37,38]. After adjusted for related covariates, the associations between adiposity (WC and WHtR) and the status of 25(OH)D in Orang Asli male adults remained almost constant and significant. These results suggested that the reported associations in Orang Asli men were prominent. Several studies have reported similar findings [36,37,39,40]. Gonzalez et al. [36,39] reported that men who had WC ≥102 cm and WHtR > 0.5 had significantly lower serum 25(OH)D concentrations than their counterparts. Among Portuguese older male adults [37,40], those with WC > 102 cm had higher odds of being at risk of deficient (<30 nmol/L) and insufficient (30 to <50 nmol/L) 25(OH)D concentration, as compared to men with WC ≤ 94 cm. The present study applied a cut-off of 90 cm to indicate at-risk WC which was lower that other reported studies as this cut-off value is deemed to be associated with risk of adverse health outcomes in Asian population [28,30].

While the present findings showed WC and WHtR as indicators of obesity that were significantly associated with the status of 25(OH)D in men, the associations in women were not significant. Contrary to the current results, several studies reported that the negative associations between 25(OH)D level and obesity indicators, including WC, were found only in women [41,42]. It is unclear that there are in fact significant sex differences in the associations of 25(OH)D with obesity in different populations. Pourshahidi [43] reported that there are four proposed mechanisms that may explain a low vitamin D status in obesity, including (1) decreased sun exposure in obese individuals, (2) negative feedback from increased circulating 1, 25(OH)D concentration in obesity, (3) sequestration of vitamin D in adipose tissue, and (4) the effect of volumetric dilution. However, within these mechanisms, none of significant sex difference has been reported.

The variations in the aforementioned findings might be most likely attributable to the significant heterogeneity between the studies. In women, other than lifestyle factors, hormone levels may link obesity to vitamin D deficiency. The decline in estrogen production was thought to increase the abdominal fat [44] as well as decrease the activity of 1-α-hydroxylase, which was responsible for the activation of vitamin D, and promote the vitamin D deficiency [45] among menopausal women, as compared to women of reproductive age. As the variable of menopausal status was not assessed, this study was unable to adjust the effect of menopausal status in female adults. 

Although suboptimal 25(OH)D concentration was associated with higher odds for each BMI ≥ 30 and at-risk WHR in men but lower odds in women, the associations were not significant. Previous findings on the association between BMI and vitamin D were inconsistent. A meta-analysis demonstrated an inverse relationship between vitamin D status and BMI in adults [46], while other studies showed no significant association between the two variables [13,16]. BMI is known as a rather poor indicator of body fatness since it does not distinguish between muscle mass and adipose tissue. Men and women have a different body composition. In general, men tend to have more lean muscle mass and less body fat than women even when both of them have the same BMI. Physical inactivity and advanced age are associated with decrease in muscle mass and may result in BMI misclassification as normal weight despite having higher level of body fat. Considering these limitations, it is deduced that the predictability of body fatness by BMI on the study subjects will influence the association between BMI and vitamin D. As compared to WC, the interpretation on the association between WHR and vitamin D status is more complex. An increase in WHR can be either due to the increase of abdominal fat or decrease in lean muscle mass around the hip. The former might have a greater impact on vitamin D status since vitamin D is sequestered in the excess adipose tissue, leading to less bioavailability. Hence, findings on the association between WHR and vitamin D status might vary across the studies.

Vitamin D metabolism involves three steps, including 25-hydroxylation, 1α-hydroxylation, and 24-hydroxylation, that are performed by cytochrome P450 mixed-function oxidases (CYPs) [47]. Recent studies showed that the regulation of these vitamin D metabolizing enzymes may relate to vitamin D metabolism and further play a potential role in enhancing the regulation of metabolic processes [48]. The cholesterol side-chain cleavage enzyme (CYP11A1), which catalyzes the first step in steroidogenesis, has been found to involve in the sequential hydroxylation, predominantly at C-20 or C-22, in the vitamin D metabolism [49]. Importantly, CYP11A1-derived vitamin D metabolites display anti-proliferative, differentiation and anti-inflammatory activities and thus play a role in the regulation of physiological process and related pathology.

Hyperlipidemia is an increasing health problem which is commonly due to diet and lifestyle. Two mechanisms in which vitamin D regulates lipid metabolism have been proposed in previous studies [50,51]. Asano et al. [50] suggested that 25(OH)D might act as an endogenous inhibitor of sterol regulatory element-binding proteins (SREBPs) that are required for the synthesis and uptake of cholesterol and fatty acids in the body. The activated SREBPs increase the gene expression that create enzymes needed to make lipids. As 25(OH)D might impair the activation of SREBPs, it could eventually control the lipid production. Meanwhile, Li et al. [51] proposed that vitamin D deficiency might increase cholesterol concentration by enhancing cholesterol biosynthesis, rather than by reducing its catabolism. Vitamin D deficiency could raise hepatic cholesterol biosynthesis that is involved in the reduction of transcriptional activity of VDR, downregulation of insulin-induced gene-2 (INSIG2) expression and alteration of INSIG-2/SREBP-2 pathway. It is unclear whether the underlying mechanisms differ by sex, adiposity and lipid parameters.

Findings of the present study revealed that the association between suboptimal 25(OH)D concentration and poor lipid profile was more apparent in terms of LDL-C. Results showed that suboptimal 25(OH)D concentration was associated with higher odds for high LDL-C in Orang Asli adults and the association was found to be significant in men but not women. While alcohol consumption was significantly associated with LDL-C level in men (Table S1), the association between LDL-C and vitamin D status remained significant after adjusting for alcohol status. Zhang et al. [52] reported similar findings, whereby a significant difference in LDL-C (not TC, TG and HDL-C) was shown in men among the three vitamin D categories (deficiency, insufficiency and sufficiency). LDL-C is considered by NCEP as the primary target for cholesterol lowering therapy for the prevention of CVD. The prominent association between LDL-C and 25(OH)D, as shown in this study, might be useful for future studies to further explore the role of vitamin D in the CVD prevention in this population.

This study did not rule out the association between vitamin D status and lipid profile in Orang Asli adults, even though most of the associations between suboptimal 25(OH)D concentration and lipid parameters were not significant. A total of 31.2% of Orang Asli adults in the present study were with 25(OH)D concentration >100 nmol/L. Among these adults, 8.1%, 17.3%, 6.9%, and 10.4% of them were with high TC, TG, and LDL-C as well as low HDL-C, respectively. The results might imply that the relationship between 25(OH)D concentration and poor lipid profile among Orang Asli was not inversely linear. Further examination of the differences in serum lipid among female adults by 3 categories of 25(OH)D concentration (<75 nmol/L, 75–100 nmol/L and >100 nmol/L) showed that Orang Asli women with 25(OH)D concentration >100 nmol/L had significantly higher TG and lower HDL-C than women with 25(OH)D concentration <75 nmol/L (Table S2). To note, previous studies [53,54] reported reverse J-shaped or U-shaped associations between serum 25(OH)D concentration with several health outcomes including CVD and all-cause mortality with a serum level above 100 nmol/L was shown to be significantly associated to higher all-cause mortality. Therefore, future studies to explore the associations between elevated level of 25(OH)D concentration and adverse health outcomes in this population is necessary.

While vitamin D deficiency is a global health problem [55], it is less common in Orang Asli adults in this study. The mean serum 25(OH)D concentration (89.53 ± 23.01 nmol/L) in this study population was much higher than the reported values of other ethnic groups in Malaysia in which the mean/median 25(OH)D concentrations ranged from 54 to 64 nmol/L and 36.2 to 68.8 nmol/L in men and women, respectively [23,24,25,26,27]. In addition, the prevalence of vitamin D deficiency (25 (OH)D concentration <50 nmol/L) in Orang Asli adults (1.4%) was much lower than other Malaysian adults (ranged from 12.2% to as high as 80.9%). Although the Orang Asli adults seemed to have better vitamin D status as compared to indigenous peoples residing in other countries [56,57,58], it should be noted that the comparison is limited as key characteristics such as latitude of residence may differ between groups. Living near to the equator allows Orang Asli adults, who usually spend more time outdoor, to receive more sunlight throughout the year and it may enhance the cutaneous synthesis of vitamin D.

The strength of this study is the relatively comprehensive lipid and adiposity parameters measured. However, several limitations inherent to the present study could have influenced the findings. The measurement of 25(OH)D concentration was based on a one-time measure and 25(OH)D concentration is assumed to remain almost constant at different time periods. Although, there may be a bidirectional relationship between the 25(OH)D concentration and adiposity measures, this study presumed that the relationship was only in one direction. Interactions between parathyroid hormone and levels of vitamin D are often discussed in the literature. However, as the measurement of serum parathyroid hormone (PTH) was not included in the study, we were unable to determine whether the associations between serum 25(OH)D concentration with adiposity and lipid profile were caused by secondary hyperparathyroidism. The number of men with suboptimal vitamin D status was relatively small and this could limit certainty of the results. As the present study was cross-sectional in design, the causal relationships of 25(OH)D concentration with obesity and lipid profile cannot be inferred. Due to lack of representative study sample, the results of this study could not be generalized to other Orang Asli sub-tribes. Although the present study adjusted for prominent confounding factors, there could be other factors that were not considered in the statistical analysis because the data were not collected. Thus, there is a possibility for residual confounding.

## 5. Conclusions

It seems paradoxical that a high proportion of Orang Asli men and women were with poor lipid profile and central obesity, respectively, despite having relatively normal 25(OH)D concentration. Men with suboptimal 25(OH)D concentration were more likely to have at-risk WC, WHtR and LDL-C. The sex-specific associations between 25(OH)D concentration with adiposity measures and lipid profile observed in this Orang Asli population warrant further investigation.

## Figures and Tables

**Table 1:** Characteristics of respondents by sex.

Characteristics	Mean ± SD *n* (%)		
	Men (*n* = 164)	Women (*n* = 391)	Total (*N* = 555)
**Age (years)**	40.6 ± 13.6	36.4 ± 12.7	37.6 ± 13.1
18–30	46 (28.0)	149 (38.1)	195 (35.1)
31–40	40 (24.4)	112 (28.6)	152 (27.4)
41–50	38 (23.2)	76 (19.4)	114 (20.5)
>50	40 (24.4)	54 (13.8)	94 (17.0)
Education (years)	4.6 ± 3.8	4.0 ± 4.2	4.2 ± 4.1
No formal education	39 (23.8)	148 (37.9)	187 (33.7)
Primary	85 (51.8)	140 (35.8)	225 (40.5)
Secondary	37 (22.6)	93 (23.8)	130 (23.4)
Tertiary	3 (1.8)	10 (2.6)	13 (2.4)
**Employment/Occupation**			
Employed			
Rubber tapper	127 (77.4)	170 (43.5)	297 (53.5)
Others ^1^	25 (15.4)	24 (6.1)	49 (8.8)
Unemployed			
Housewife	-	143 (36.6)	143 (25.8)
Others (Student/Retired/Jobless)	12 (7.3)	54 (13.8)	66 (11.9)
**Involvement in traditional lifestyle (over the past one year)**			
Gathering			
Daily	11 (6.7)	6 (1.5)	17 (3.1)
Weekly	28 (17.1)	44 (11.3)	72 (13.0)
Monthly	25 (15.2)	29 (7.4)	54 (9.7)
Never	100 (61.0)	312 (79.8)	412 (74.2)
Hunting			
Daily	-	-	-
Weekly	10 (6.1)	2 (0.5)	12 (2.1)
Monthly	11 (6.7)	5 (1.3)	16 (2.9)
Never	143 (87.2)	384 (98.2)	527 (95.0)
Ever—(former ^2^ and current) smoker	125 (76.2)	163 (41.7)	288 (51.9)
Ever—(former and current) drinker	84 (51.2)	11 (2.8)	95 (17.1)
**Self-reported average time spent (hour/day)**			
in sitting down	3.20 ± 1.89	3.75 ± 2.54	3.59 ± 2.38
in sun	6.38 ± 1.31	4.72 ± 1.74	5.21 ± 1.79

^1^ Others (e.g., Oil palm plantation workers, construction workers).^2^ Former smoker/drinker: Had smoked/consumed alcohol during a whole year, had not smoked/consumed alcohol during the last month and stopped smoking/drinking.

**Table 2:** Anthropometric and biochemical variables of respondents by sex.

Variables	Mean ± SD *n* (%)		
	Men (*n* = 164)	Women (*n* = 391)	Total (*N* = 555)
25(OH)D concentration (nmol/L)	99.65 ± 23.32	85.28 ± 21.54	89.53 ± 23.01
Insufficiency (50 to <75)	17 (10.4)	121 (30.9)	138 (24.9)
Deficiency (<50)	2 (1.2)	6 (1.5)	8 (1.4)
Anthropometry			
BMI ^1^ (kg/m^2^)	23.89 ± 4.01	23.84 ± 4.76	23.86 ± 4.55
Overweight (25.0–29.9)	36 (22.0)	108 (27.7)	144 (25.9)
Obese (≥30.0)	20 (12.2)	38 (9.7)	58 (10.5)
Waist circumference ^2^ (cm)	79.82 ± 11.96	76.0 ± 11.67	77.0 ± 11.84
At risk (M: ≥90; W: ≥80)	32 (19.5)	135 (34.5)	167 (30.1)
Waist-height ratio ^3^(WHtR)	0.50 ± 0.07	0.51 ± 0.08	0.51 ± 0.07
At risk (≥0.50)	73 (44.5)	219 (56.0)	292 (52.6)
Waist–hip ratio ^1^ (WHR)	0.90 ± 0.10	0.85 ± 0.08	0.86 ± 0.09
At risk (M: ≥0.9; W: ≥0.85)	76 (46.3)	192 (49.1)	268 (48.3)
Percent body fat ^4^ (%BF)	22.10 ± 6.07	28.65 ± 6.80	26.72 ± 7.23
High (M: ≥25; W: ≥32)	52 (31.7)	124 (31.7)	176 (31.7)
Lipid parameters ^5^			
Total cholesterol (TC) (mmol/L)	5.10 ± 1.15	4.81 ± 0.89	4.89 ± 0.98
High (≥6.2)	26 (15.9)	31 (7.9)	57 (10.3)
Triglycerides (TG) ^6^ (mmol/L)	1.70 (1.30, 2.50)	1.30 (0.90, 1.80)	1.40 (1.00, 2.00)
High (≥2.3)	50 (30.5)	56 (14.3)	106 (19.1)
High density lipoprotein cholesterol (HDL-C) (mmol/L)	1.23 ± 0.29	1.38 ± 0.32	1.34 ± 0.31
Low (<1.0)	26 (15.9)	24 (6.1)	50 (9.0)
Low density lipoprotein cholesterol (LDL-C) (mmol/L)	2.95 ± 0.93	2.74 ± 0.76	2.80 ± 0.82
High (≥4.1)	23 (14.0)	25 (6.4)	48 (8.6)

Classification based on ^1^ WHO, 2000; ^2^ International Diabetes Federation, 2006; ^3^ Browning, Hsieh and Ashwell, 2010; ^4^ Nieman, 2003; ^5^ NCEP ATP (III), 2002; ^6^ Data expressed as median (25th percentile, 75th percentile).

**Table 3:** Characteristics of respondents by serum 25(OH)D concentration (*N* = 555) †.

Characteristics	Sufficient 25(OH)D Concentration (≥75 nmol/L)			Suboptimal 25(OH)D Concentration (<75 nmol/L)			*p*-Value *		
	Men (*n* = 145)	Women (*n* = 264)	Overall (*n* = 409)	Men (*n* = 19)	Women (*n* = 127)	Overall (*n* = 146)	Men	Women	Overall
**Age (years)**	40.4 ± 13.7	37.0 ± 12.2	38.3 ± 12.8	42.3 ± 13.1	35.1 ± 13.8	36.1 ± 13.9	0.573	0.187	0.096
**Weight (kg)**	60.82 ± 11.81	52.85 ± 12.24	55.67 ± 12.66	64.79 ± 10.81	53.87 ± 12.60	55.29 ± 12.88	0.166	0.445	0.755
**Height (cm)**	159.85 ± 6.35	149.10 ± 5.37	152.91 ± 7.70	160.29 ± 5.61	148.54 ± 6.65	150.07 ± 7.62	0.774	0.377	0.000
**BMI ^1^**	23.72 ± 4.02	23.66 ± 4.75	23.68 ± 4.50	25.19 ± 3.82	24.23 ± 4.76	24.36 ± 4.65	0.134	0.265	0.123
Underweight (<18.5)	10 (6.9)	28 (10.6)	38 (9.3)	1 (5.3)	13 (10.2)	14 (9.6)			
Normal (18.5–24.9)	90 (62.1)	145 (54.9)	235 (57.5)	7 (36.8)	59 (46.5)	66 (45.2)			
Overweight (25.0–29.9)	28 (19.3)	66 (25.0)	94 (23.0)	8 (42.1)	42 (33.1)	50 (34.2)			
Obese (≥30.0)	17 (11.7)	25 (9.5)	42 (10.3)	3 (15.8)	13 (10.2)	16 (11.0)			
**WC (cm) ^2^**	78.32 ± 11.73	75.44 ± 11.35	76.46 ± 11.55	86.57 ± 11.50	77.29 ± 12.28	78.50 ± 12.54	0.004	0.142	0.074
Normal (M: <90; W: <80)	121 (83.4)	177 (67.0)	298 (72.9)	11 (57.9)	79 (62.2)	90 (61.6)			
At risk (M: ≥90; W: ≥80)	24 (16.6)	87 (33.0)	111 (27.1)	8 (42.1)	48 (37.8)	56 (38.4)			
**Waist-height ratio (WHtR) ^3^**	0.49 ± 0.07	0.51 ± 0.07	0.50 ± 0.07	0.54 ± 0.07	0.52 ± 0.08	0.52 ± 0.08	0.007	0.071	0.002
Normal (<0.50)	86 (59.3)	122 (46.2)	208 (50.9)	5 (26.3)	50 (39.4)	55 (37.7)			
At risk (≥0.50)	59 (40.7)	142 (53.8)	201 (49.1)	14 (73.7)	77 (60.6)	91 (62.3)			
**Hip circumference (cm)**	87.67 ± 8.46	88.97 ± 9.77	88.51 ± 9.34	93.71 ± 8.26	90.58 ± 9.49	90.98 ± 9.37	0.004	0.124	0.006
**Waist–hip ratio (WHR) ^1^**	0.89 ± 0.10	0.85 ± 0.07	0.86 ± 0.08	0.92 ± 0.07	0.85 ± 0.09	0.86 ± 0.09	0.317	0.324	0.775
Normal (M: <0.9; W: <0.85)	81 (55.9)	131 (49.6)	212 (51.8)	7 (36.8)	68 (53.5)	75 (51.4)			
At risk (M: ≥0.9; W: ≥0.85)	64 (44.1)	133 (50.4)	197 (48.2)	12 (63.2)	59 (46.5)	71 (48.6)			
**Percent body fat (%BF) ^4^**	21.75 ± 5.96	28.59 ± 6.70	26.17 ± 7.22	24.76 ± 6.40	28.78 ± 7.02	28.26 ± 7.05	0.042	0.156	0.003
Low	-	-	-	-	1 (0.8)	1 (0.7)			
Acceptable (lower end)	22 (15.1)	65 (24.6)	87 (21.3)	2 (10.5)	23 (18.1)	25 (17.1)			
Acceptable (upper end)	81 (55.9)	117 (44.3)	198 (48.4)	7 (36.8)	61 (48.0)	68 (46.6)			
High (M: ≥25; W: ≥32)	42 (29.0)	82 (31.1)	124 (30.3)	10 (52.6)	42 (33.1)	52 (35.6)			
**TC ^5^ (mmol/L)**	5.02 ± 1.12	4.75 ± 0.92	4.84 ± 1.00	5.73 ± 1.23	4.93 ± 0.81	5.04 ± 0.91	0.011	0.048	0.038
Desirable (<5.2)	78 (53.8)	192 (72.7)	270 (66.0)	7 (36.8)	84 (66.1)	91 (62.3)			
Borderline high (5.2–6.1)	46 (31.7)	49 (18.6)	95 (23.2)	7 (36.8)	35 (27.6)	42 (28.8)			
High (≥6.2)	21 (14.5)	23 (8.7)	44 (10.8)	5 (26.4)	8 (6.3)	13 (8.9)			
**Triglycerides (TG) ^5^ (mmol/L)**	1.70 ± 1.00	1.30 ± 0.88	1.40 ± 0.93	2.00 ± 1.52	1.10 ± 0.66	1.20 ± 0.91	0.098	0.001	0.000
Normal (<1.7)	72 (49.7)	177 (67.0)	249 (60.9)	6 (31.6)	95 (74.8)	101 (69.2)			
Borderline high (1.7–2.2)	30 (20.7)	44 (16.7)	74 (18.1)	6 (31.6)	19 (15.0)	25 (17.1)			
High (≥2.3)	43 (29.6)	43 (16.3)	86 (21.0)	7 (36.8)	13 (10.2)	20 (13.7)			
**HDL-C ^5^ (mmol/L)**	1.23 ± 0.30	1.35 ± 0.31	1.31 ± 0.31	1.28 ± 0.25	1.44 ± 0.32	1.42 ± 0.31	0.426	0.004	0.000
Low (<1.0)	25 (17.2)	18 (6.8)	43 (10.5)	1 (5.3)	6 (4.7)	7 (4.8)			
Normal (1.0–1.5)	105 (72.5)	176 (66.7)	281 (68.7)	15 (78.9)	78 (61.4)	93 (63.7)			
High (≥1.6)	15 (10.3)	70 (26.5)	85 (20.8)	3 (15.8)	43 (33.9)	46 (31.5)			
**LDL-C ^5^ (mmol/L)**	2.92 ± 0.94	2.67 ± 0.77	2.76 ± 0.84	3.25 ± 0.82	2.88 ± 0.74	2.92 ± 0.75	0.175	0.013	0.045
Optimal (<2.6)	52 (35.9)	124 (47.0)	176 (43.0)	3 (15.8)	39 (30.7)	42 (28.8)		
Above optimal (2.6–3.3)	42 (29.0)	92 (34.8)	134 (32.8)	7 (36.8)	57 (44.9)	64 (43.8)		
Borderline high (3.4–4.0)	35 (24.1)	31 (11.7)	66 (16.1)	2 (10.5)	23 (18.1)	25 (17.1)		
High (≥4.1)	16 (11.0)	17 (6.4)	33 (8.1)	7 (36.8)	8 (6.3)	15 (10.3)		

^†^ Data expressed as mean ± SD except for TG (median ± SD); * Differences between individuals with sufficient and suboptimal 25(OH) D concentration; ^1^ WHO, 2000; ^2^ International Diabetes Federation, 2006; ^3^ Browning, Hsieh and Ashwell, 2010; ^4^ based on Nieman (2003): Low (M: < 6; W: 9), Acceptable (lower end) (M: 6-<16; W: 9-<24), Acceptable (upper end): (M:16-<25; W: 24-<32); ^5^ NCEP ATP (III), 2002.

**Table 4:** Association between 25 (OH) D concentration level and obesity status in men and women.

25(OH) D Concentration Level		Obesity				
		BMI ≥ 30 kg/m^2^	WC ≥ 90 cm(M); ≥80 cm (W)	WHtR ≥ 0.50	WHR ≥0.9 (M); ≥0.85 (W)	%BF ≥ 25 (M); ≥32 (W)
		Odds Ratio (95% CI)				
**Men**	**Model 1**					
	Sufficient	1.0	1.0	1.0	1.0	1.0
	Suboptimal	1.41 (0.37, 5.35)	3.67 (1.34, 10.07) *	4.08 (1.40, 11.94) *	2.17 (0.81, 5.83)	2.73 (1.03, 7.18) *
	**Model 2**					
	Sufficient	1.0	1.0	1.0	1.0	1.0
	Suboptimal	1.38 (0.36, 5.26)	3.68 (1.34, 10.13) *	4.02 (1.36, 11.84) *	2.10 (0.77, 5.78)	2.75 (0.99, 7.64)
	**Model 3**					
	Sufficient	1.0	1.0	1.0	1.0	1.0
	Suboptimal	1.25 (0.31, 4.95)	4.07 (1.42, 11.69) *	4.10 (1.36, 12.15) *	2.13 (0.76, 5.94)	2.83 (1.00, 8.00)
Women	**Model 1**					
	Sufficient	1.0	1.0	1.0	1.0	1.0
	Suboptimal	1.09 (0.54, 2.21)	1.24 (0.80, 1.92)	1.32 (0.86, 2.04)	0.86 (0.56, 1.31)	1.10 (0.70, 1.72)
	**Model 2**					
	Sufficient	1.0	1.0	1.0	1.0	1.0
	Suboptimal	1.00 (0.49, 2.05)	1.23 (0.79, 1.91)	1.38 (0.89, 2.13)	0.92 (0.59, 1.42)	1.10 (0.70, 1.72)
	**Model 3**					
	Sufficient	1.0	1.0	1.0	1.0	1.0
	Suboptimal	0.95 (0.46, 1.96)	1.15 (0.73, 1.82)	1.36 (0.88, 2.12)	0.92 (0.59, 1.44)	1.05 (0.66, 1.66)

A 25(OH) D concentration <75 nmol/L is an indication of suboptimal vitamin D concentration; BMI: Body Mass Index; WC: Waist circumference;.WHtR: Weight–height ratio; WHR: Waist–hip ratio; %BF: Percent body fat; Model 1: unadjusted; Model 2: adjusted for age; Model 3: adjusted for age, time spent in sitting down (hours/day), smoking status (1) (1 = ever smoker; 0 = non-smoker), drinking status (1 = ever-drinker; 0 = non-drinker).* *P* < 0.05.

**Table 5:** Association between 25 (OH) D concentration level and lipid status in men and women.

25(OH) D Concentration Level		Poor Lipid Profile			
		TC ≥ 6.2 mmol/L	TG ≥ 2.3 mmol/L	HDL-C < 1.0 mmol/L	LDL-C ≥ 4.1 mmol/L
		Odds Ratio (95% CI)			
**Men**	**Model 1**				
	Sufficient	1.0	1.0	1.0	1.0
	Suboptimal	2.11 (0.69, 6.47)	1.38 (0.51, 3.75)	0.27 (0.03, 2.09)	4.70 (1.62, 13.67) *
	**Model 2**				
	Sufficient	1.0	1.0	1.0	1.0
	Suboptimal	2.06 (0.67, 6.35)	1.37 (0.50, 3.71)	0.26 (0.03, 2.02)	4.63 (1.58, 13.58) *
	**Model 3**				
	Sufficient	1.0	1.0	1.0	1.0
	Suboptimal	1.68 (0.52, 5.45)	0.81 (0.27, 2.42)	0.23 (0.03, 1.89)	4.53 (1.46, 14.02) *
	**Model 4**				
	Sufficient	1.0	1.0	1.0	1.0
	Suboptimal	1.67 (0.51, 5.50)	0.79 (0.25, 2.36)	0.25 (0.03, 2.06)	5.03 (1.52, 16.71) *
Women	**Model 1**				
	Sufficient	1.0	1.0	1.0	1.0
	Suboptimal	0.70 (0.31, 1.62)	0.59 (0.30, 1.13)	0.68 (0.26, 1.75)	0.98 (0.41, 2.33)
	**Model 2**				
	Sufficient	1.0	1.0	1.0	1.0
	Suboptimal	0.72 (0.31, 1.68)	0.60 (0.31, 1.16)	0.66 (0.26, 1.71)	1.01 (0.42, 2.42)
	**Model 3**				
	Sufficient	1.0	1.0	1.0	1.0
	Suboptimal	0.71 (0.30, 1.67)	0.50 (0.29, 1.02)	0.59 (0.23, 1.55)	1.04 (0.43, 2.51)
	**Model 4**				
	Sufficient	1.0	1.0	1.0	1.0
	Suboptimal	0.73 (0.31, 1.72)	0.51 (0.29, 1.03)	0.58 (0.22, 1.55)	1.05 (0.43, 2.53)

A 25(OH) D concentration <75 nmol/L is an indication of suboptimal vitamin D concentration; TC: Total cholesterol; TG: Triglycerides; HDL-C: High density lipoprotein cholesterol; LDL-C: Low density lipoprotein cholesterol; Model 1: unadjusted; Model 2: adjusted for age; Model 3: adjusted for age and adiposity (WC and %BF). Model 4: adjusted for age, adiposity (WC and BF%), time spent in sitting (hours/day), smoking status (1) (1 = ever smoker; 0 = non-smoker), drinking status (1 = ever-drinker; 0 = non-drinker), * *P* < 0.05.

## Supplementary Materials

The following are available online at https://www.mdpi.com/1660-4601/17/8/2855/s1, Table S1: Association between alcohol consumption and LDL-C level in men; Table S2: Differences in serum lipid among the 3 categories of 25(OH)D concentration in women.
