# Supplementary material for: Associations of Serum 25-Hydroxyvitamin D with Adiposity and At-Risk Lipid Profile Differ for Indigenous (Orang Asli) Male and Female Adults of Peninsular Malaysia
Source: Int J Environ Res Public Health. 2020 Apr 21;17(8):2855. doi: 10.3390/ijerph17082855 (PMC7215365; doi:10.3390/ijerph17082855)
Supplement: Supplementary file 1 [file ijerph-17-02855-s001.pdf]

International Journal of
Environmental Research and Public Health

Supplementary Material

Table 1. Association between alcohol consumption and LDL-C level in men.

**Alcohol consumption**	**LDL-C level**		**χ²**	***p*-value**
	**<4.1 mmol/L**	**≥4.1 mmol/L**		
Ever- drinker	77 (91.7)	7 (8.3)	4.63	0.031
Non drinker	64 (80.0)	16 (20.0)		

Table 2. Differences in serum lipid among the 3 categories of 25 (OH)D concentration in women.

**Serum lipid**	**25 (OH)D concentration**			***F*-value**	***p*-value**
	**<75 nmol/L**	75-100 nmol/L	>100 nmol/L		
TC	4.94 ± 0.81 [a]	4.82 ± 0.92 [b]	4.58 ± 0.91 [ab]	4.17	0.016
TG	1.32 ± 0.66 [a b]	1.60 ± 0.89 [a]	1.55 ± 0.86 [b]	4.52	0.011
HDL-C	1.44 ± 0.32 [a b]	1.34 ± 0.32 [a]	1.36 ± 0.29 [b]	4.20	0.016
LDL-C	2.88 ± 0.74 [a]	2.75 ± 0.79 [b]	2.52 ± 0.72 [ab]	6.01	0.003

For post-hoc results (LSD tests): [a b] Significant difference between the 2 categories, $p<0.05$.
